# Childhood Lifestyle Behaviors and Mental Health Symptoms in Adolescence

**DOI:** 10.1001/jamanetworkopen.2024.60012

**Published:** 2025-02-14

**Authors:** Eero A. Haapala, Marja H. Leppänen, Silja Kosola, Kaija Appelqvist-Schmidlechner, Siiri-Liisi Kraav, Juuso J. Jussila, Tommi Tolmunen, David R. Lubans, Aino-Maija Eloranta, Ursula Schwab, Timo A. Lakka

**Affiliations:** 1 Sports and Exercise Medicine, Faculty of Sport and Health Sciences, University of Jyväskylä, Jyväskylä, Finland; 2 Institute of Biomedicine, School of Medicine, University of Eastern Finland, Kuopio, Finland; 3 Faculty of Medicine, Clinicum, University of Helsinki, Helsinki, Finland; 4 Research, Development and Innovations, Western Uusimaa Wellbeing Services County, Finland; 5 Finnish Institute for Health and Welfare, Helsinki, Finland; 6 Department of Social Sciences, Faculty of Social Sciences and Business Studies, University of Eastern Finland, Kuopio, Finland; 7 Institute of Public Health and Clinical Nutrition, School of Medicine, University of Eastern Finland, Kuopio, Finland; 8 Department of Adolescent Psychiatry, Kuopio University Hospital, Kuopio, Finland; 9 Centre for Active Living and Learning, College of Human and Social Futures, University of Newcastle, Callaghan, New South Wales, Australia; 10 Hunter Medical Research Institute, New Lambton Heights, New South Wales, Australia; 11 Department of Medicine, Endocrinology and Clinical Nutrition, Kuopio University Hospital, Kuopio, Finland; 12 Department of Clinical Physiology and Nuclear Medicine, Kuopio University Hospital, Kuopio, Finland; 13 Foundation for Research in Health Exercise and Nutrition, Kuopio Research Institute of Exercise Medicine, Kuopio, Finland; 14 Institute of Clinical Medicine/Psychiatry, University of Eastern Finland, Kuopio, Finland

## Abstract

**Importance:**

Mental health problems often arise during adolescence and early adulthood, affecting up to 25% to 30% of young people. Enhancing the ability to identify children and adolescents at increased risk of mental health problems and uncover factors that promote mental health from childhood to adolescence is important.

**Objective:**

To investigate if cumulative lifestyle behaviors from childhood to adolescence are associated with perceived stress and depressive symptoms in adolescence.

**Design, Setting, and Participants:**

Prospective cohort study in Finland using baseline data collected between October 2007 and November 2009 and 8-year follow-up data collected between December 2015 and December 2017 as part of the Physical Activity and Nutrition in Children (PANIC) study of children aged 6 to 9 years. Data were analyzed from January to February 2024.

**Exposures:**

Cumulative physical activity (PA), sedentary behavior (SB), sleep, and diet quality from childhood to adolescence. PA and screen time (ST) assessed by a questionnaire; PA at different intensities, SB, and sleep duration assessed by a combined movement and heart rate sensor; diet quality assessed by 4-day food records and the Baltic Sea Diet Score computed at baseline, 2-year follow-up, and 8-year follow-up examinations.

**Main Outcomes and Measures:**

Perceived stress assessed by the Finnish version of the Cohen Perceived Stress Scale, and depressive symptoms by the Beck Depression Inventory at 8-year follow-up examinations. Linear regression analyses were used to evaluate associations between cumulative lifestyle behaviors over 8 years with the outcomes.

**Results:**

Altogether, 187 adolescents (97 boys [51.9%]; mean age, 15.8 [0.4] years) had valid data on self-reported lifestyle behaviors, and 170 adolescents had valid data on device-assessed lifestyle behaviors. Perceived stress scores ranged from 2 to 33 and depressive symptoms scores from 0 to 31. Self-reported total PA and supervised exercise were inversely associated with perceived stress (standardized regression coefficient [β] = −0.15; 95% CI, −0.31 to −0.01 and β = −0.15; 95% CI, −0.29 to −0.01, respectively) and depressive symptoms (β = −0.17; 95% CI, −0.31 to −0.02 and β = −0.14; 95% CI, −0.29 to −0.0, respectively). Total ST (β = 0.27; 95% CI, 0.13 to 0.41), computer use (β = 0.16; 95% CI, 0.01 to 0.30), and mobile device use (β = 0.28; 95% CI, 0.16 to 0.41) were positively associated with perceived stress. Total ST (β = 0.30; 95% CI, 0.15 to 0.44) and mobile device use (β = 0.33; 95% CI, 0.19 to 0.46) were positively associated with depressive symptoms.

**Conclusions and Relevance:**

This cohort study of Finnish children and adolescents found that higher PA and lower ST from childhood were associated with perceived stress and depressive symptoms in adolescence. These findings emphasize reducing screen time and increasing PA to promote mental health in youth.

## Key Points

**Question:**

Are cumulative lifestyle behaviors, including physical activity, sedentary behavior, sleep, and diet quality from childhood to adolescence, associated with perceived stress and depressive symptoms in adolescence?

**Findings:**

In this cohort study of 187 adolescents, those with a higher cumulative exposure to self-reported total screen time and particularly mobile device use from childhood had higher perceived stress and depressive symptoms scores. Self-reported total physical activity and supervised exercise were inversely associated with perceived stress and depressive symptoms.

**Meaning:**

These findings underscore the significance of reducing screen time and increasing physical activity to promote mental health in youth.

## Teaser

This cohort study evaluates whether cumulative lifestyle behaviors from childhood to adolescence are associated with perceived stress and depressive symptoms in adolescence.

## Introduction

Mental health problems, particularly depression and anxiety, are among the leading causes of disability globally,^1^ affecting 25% to 30% of adolescents and young adults.^2^ Mental health problems often arise during adolescence and early adulthood.^3^ They are the primary cause of sickness allowance in Finnish adolescents and young adults.^4^ Therefore, identifying protective and risk factors for these disorders and targeting preventive actions among those at increased risk is crucial.

Healthy lifestyle behaviors, including high physical activity (PA) and low sedentary behavior (SB) levels, as well as sufficient sleep, have been recognized as potentially modifiable factors alleviating mental health symptoms.^5,6^ Regular PA is associated with a decreased risk of depression in adults, with even relatively low PA doses offering benefits.^7,8^ Most previous studies in children and adolescents have been cross-sectional with mixed results.^9,10^ PA interventions in youth have shown modest improvements in^9^ or no effects^11^ on mental health symptoms. These mixed findings suggest that contextual factors may act as moderators.^5^ Although sports participation may have the greatest favorable associations with mental health outcomes,^9,12^ unsupervised PA can also benefit mental health.^5^ However, characteristics of PA that may contribute to mental health, such as type, intensity, volume, contexts, and delivery, remain unclear.^9,10^

Although the evidence regarding the associations of SB with mental health remains unclear,^13,14,15^ high levels of screen time (ST) have been associated with mental health problems in youth,^16,17,18^ with internet and social media use having the greatest adverse associations.^16,19^ Most previous studies have been cross-sectional, and only a few have used device-assessed SB.^9^ Frequent social media use may predict higher psychological distress among youth across 2 years,^20^ while device-assessed SB was inversely associated with hyperactivity in youth over 4 years.^15^

Sleep duration has been positively associated with mental health in children and adolescents,^21^ while sleep problems have been associated with depressive symptoms in adolescents.^22,23^ Moreover, the recommended levels of sleep and screen time show greater associations with mental health in youth than achieving the recommended daily 60 minutes of moderate-to-vigorous PA.^24^

In adults, a healthier diet has been associated with lower levels of depressive symptoms and better quality of life.^25,26^ Few cross-sectional studies have explored the association between dietary factors and mental health in children and adolescents, suggesting a negative association between poor diet quality and mental health.^27,28,29^ However, longitudinal studies have found mixed associations between diet quality and mental health in adolescents.^29,30^

Understanding the importance of different lifestyle behaviors in preventing and mitigating mental health symptoms in children and adolescents is essential for effective evidence-informed lifestyle interventions. However, most previous studies have focused on different lifestyle behaviors in isolation, and prospective studies on the associations of multiple lifestyle behaviors from childhood with mental health symptoms in adolescence are lacking. We investigated the associations of long-term exposure to a range of lifestyle behaviors, including PA, SB, sleep, and diet quality, from childhood to adolescence over 8 years with mental health in adolescence.

## Methods

Detailed methodological information is provided in the eMethods in Supplement 1. The research ethics committee of the Hospital District of Northern Savo approved the study protocol in 2006 and extended its approval until the 8-year follow-up examinations in 2015. The parents or caregivers of the children provided written informed consent, and the children assented to participation. At the 8-year follow-up, the participants reaffirmed their consent. The study was conducted in accordance with the principles of the Declaration of Helsinki as revised in 2008. The study followed the Strengthening the Reporting of Observational Studies in Epidemiology (STROBE) reporting guideline.

### Study Design and Participants

The present analyses are based on data from the Physical Activity and Nutrition in Children (PANIC) study, which is an 8-year PA and dietary intervention study in a general population of children followed up until adolescence.^31,32^ Of the children included in the study, 438 (87%) attended the 2-year follow-up examinations in 2009 to 2011, and 277 (55%) attended the 8-year follow-up examinations in 2015 to 2017. We treated the participants as a prospective cohort in the present analyses because mental health symptoms were assessed only at the 8-year follow-up.

### Assessment of Lifestyle Behaviors at Baseline, 2-Year Follow-Up, and 8-Year Follow-Up

#### Self-Reported Lifestyle Behaviors

We assessed total PA, unsupervised PA, sports participation, participation in all supervised exercise, total ST, habitual TV viewing time, computer use, and mobile device use by the PANIC Physical Activity Questionnaire. We assessed the consumption of food and drinks and the nutrient intake using food records (4 predefined consecutive days, including 2 weekdays and 2 weekend days or 3 weekdays and 1 weekend day).^32^ The Baltic Sea Diet Score (BSDS)^33^ was used as an indicator of overall diet quality.

#### Device-Assessed Lifestyle Behaviors

We used a uniaxial accelerometer with a built-in heart rate sensor (Actiheart, CamNtech Ltd) to assess PA, SB, and sleep duration. The participants were requested to wear the device continuously for a minimum of 4 consecutive days (2 weekdays and 2 weekend days).^34^ Data were accepted for analyses if at least 48 hours of activity were recorded in weekday and weekend hours, including at least 12 hours from morning (3 am-9 am), noon (9 am-3 pm), afternoon (3 pm-9 pm), and night (9 pm-3 am) to avoid potential bias from over-representing specific times and activities.^35^ We defined sedentary behavior as time spent in activity 1.5 or fewer metabolic equivalents (METs) excluding sleep and light, moderate, and vigorous PA as time spent in activity less than 1.5 and 4.0 or fewer METs, more than 4.0 and 7.0 or fewer METs, and more than 7.0 METs, respectively.

### Computation of the Measures of Cumulative Lifestyle Behaviors

We used the area under the curve (AUC) approach for lifestyle behaviors across all 3 time points, including baseline, 2-year follow-up, and 8-year follow-up to use all the data collected over the 8-year period (see eMethods in Supplement 1).^36^ The AUC describes the long-term exposure to lifestyle behaviors from childhood to adolescence.^36^ The AUCs were determined using an additive mixed model.^37^ The modeling allowed the inclusion of a nonlinear effect, which was modeled by cubic spline in addition to random intercept for individuals.^38^

### Assessment of Mental Health Symptoms at 8-Year Follow-Up

The adolescents reported their perceived stress using the Finnish version of the Cohen Perceived Stress Scale.^39^ Depressive symptoms were reported using the Beck Depression Inventory.^40^

### Assessment of Confounding Factors at 8-Year Follow-Up

Parents reported their educational degrees (vocational school or less, polytechnic, or university). Body fat percentage (BF%) was measured by the dual-energy x-ray absorptiometry (Lunar, GE Medical Systems).^41^ Pubertal status (testicular volume in boys, breast development in girls) was assessed by a research physician using a 5-stage scale described by Marshall and Tanner.^42,43^

### Statistical Analysis

Statistical analyses were performed using SPSS software version 28.0.1.1 (IBM Corp). We investigated the differences between those included in the final analyses (see eResults in Supplement 1) and those excluded and between those in the intervention and control groups using the *t* test for continuous variables and the χ^2^ test for categorical variables. Participant characteristics were described using means (SDs), median (IQRs), or percentages. The associations of cumulative lifestyle behaviors from childhood to adolescence over 8 years with mental health symptoms in adolescence were investigated using linear regression analyses adjusted for age, sex, and parental education at 8-year follow-up. The modifying effect of sex on the associations of lifestyle behaviors with mental health symptoms was studied by including a sex × lifestyle behavior interaction term in the models. The data were additionally adjusted for pubertal status and BF% at 8-year follow-up. The data on statistically significant associations were also mutually adjusted for other cumulative lifestyle behaviors. These measures were entered into the models separately to allow us to quantify their independent associations. We also adjusted the data for intervention vs control to test whether the intervention changed the associations. As it had no effect on the associations, it was not adjusted for in the final analyses. The data were reported as standardized regression coefficients (β), their 95% CIs, and corresponding *P* values. *P* values .05 or less were considered statistically significant. We considered β of 0.10 to 0.29, 0.30 to 0.49, and 0.50 or higher to describe small, medium, and large effect sizes, respectively.^44^

As a post hoc analysis based on the greatest predictors of mental health symptoms in the linear regression analyses, we divided adolescents into groups with lower or higher levels of self-reported total PA or supervised exercise (< or ≥sex-specific median) and lower or higher levels of self-reported total ST or mobile device use (< or ≥sex-specific median) from childhood to adolescence. We then compared mental health symptoms between the groups using general linear models adjusted for age, sex, and parental education and considering the Sidak correction for multiple comparisons (see eMethods, eResults, eFigures 1 and 2, and eTables 1 and 2 in Supplement 1). All statistical tests were 2-tailed. Data were analyzed from January to February 2024.

## Results

### Participants

The final study sample included 504 children (261 boys [51.8%]; age range, 6-9 years) at baseline. Altogether, 187 adolescents (mean [SD] age 15.8 [0.4] years; 97 boys [51.9%]; 68% of all adolescents at 8-year follow-up) had valid data on self-reported lifestyle behaviors and mental health symptoms, and 170 adolescents (61%) had valid data on device-assessed lifestyle behaviors and mental health symptoms (Figure). Perceived stress and depressive symptoms scores did not differ between participants in the intervention and control groups. The 187 participants included in the analyses did not differ in age, sex distribution, body mass index SD score, or BF% from those excluded, but participants came more often from families with parents having at least polytechnic degree compared those excluded (χ^2^_2_ = 7.8; *P* = .02).

**Figure.**
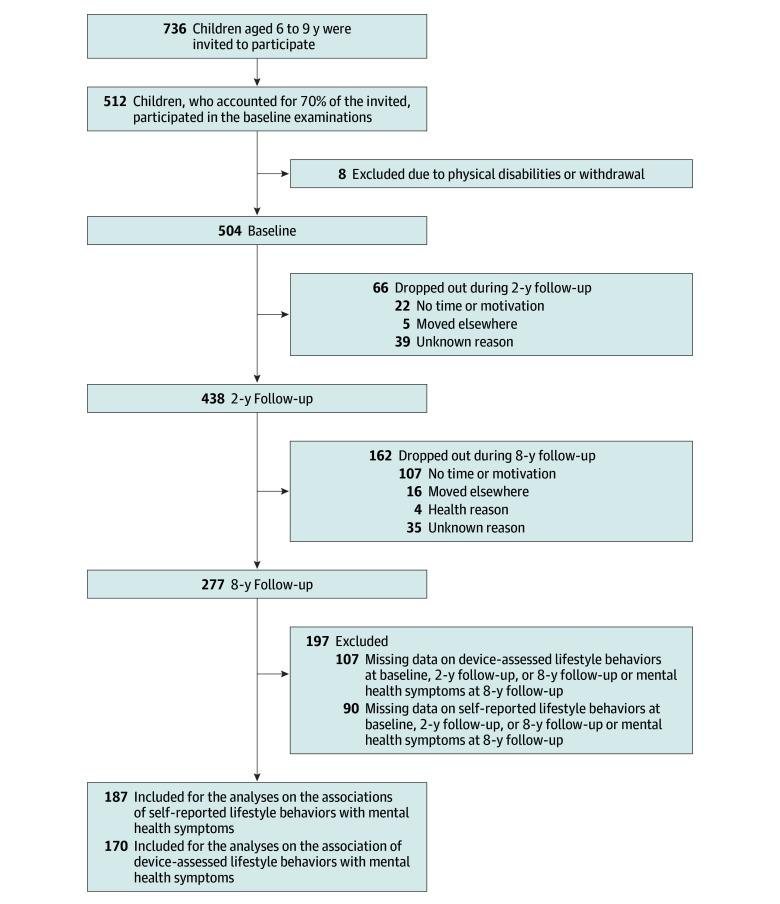
Flowchart of the Study

### Characteristics of Participants

The participants daily accumulated approximately 2 hours of self-reported total PA, approximately 0.7 hours of device-assessed moderate-to-vigorous PA, and approximately 4.7 hours of total ST and slept 9 hours per night (Table 1). The mean BSDS represented 67% of the maximal BSDS.

**Table 1.** Characteristics of Participants in Adolescence at 8-Year Follow-Up

Characteristic	Participants, No. (%)^a^		
	All (N = 187)	Girls (n = 90)	Boys (n = 97)
Age, mean (SD), y	15.8 (0.4)	15.8 (0.4)	15.8 (0.5)
Body height, mean (SD), cm	171 (8.1)	166.1 (5.8)	175.8 (7.2)
Body weight, mean (SD), kg	61.5 (11.6)	58.2 (9.4)	64.6 (12.4)
Body mass index, SD score^b^	−0.04 (1.0)	0.1 (0.8)	−0.1 (1.1)
Prevalence of overweight and obesity	24 (12.8)	10 (11.1)	14 (14.4)
Body fat percentage, median (IQR)	23.4 (23.4 to 29.7)	28.5 (24.0 to 32.2)	13.6 (10.6 to 21.1)
Pubertal status			
1	0	0	0
2	0	0	0
3	17 (9.0)	3 (3.3)	14 (14.4)
4	110 (58.8)	51 (57.0)	58 (60.0)
5	61 (32.6)	36 (40.0)	25 (25.8)
Parental education			
Vocational school or less	19 (10.3)	8 (8.9)	11 (11.3)
Polytechnic	83 (43.4)	46 (51.1)	35 (36.1)
University	86 (46.0)	36 (40.0)	51 (52.6)
Mental health symptoms			
Perceived stress score, mean (SD)	12.7 (5.9)	14.8 (6.0)	10.6 (5.1)
Depressive symptoms score, median (IQR)	1.0 (1.0 to 4.0)	2.0 (0.0 to 5.3)	1.0 (0.0 to 2.0)
Self-reported lifestyle behaviors, median (IQR)			
Total physical activity, min/d	127 (78 to 190)	101 (73 to 147)	170 (88 to 221)
Unsupervised physical activity, min/d	62 (28 to 107)	44 (23 to 78)	78 (34 to 136)
Sports, min/d	12 (0 to 47)	7 (0 to 36)	16 (0.0 60)
Supervised exercise, min/d	20 (0 to 61)	20 (4 to 40)	20 (0 to 74)
Total screen time, min/d	283 (206 to 375)	250 (194 to 323)	326 (236 to 401)
TV watching, min/d	56 (30 to 77)	56 (30 to 77)	58 (21 to 77)
Computer use, min/d	30 (0 to 99)	21 (0 to 57)	58 (5 to 143)
Mobile device use, min/d	135 (88 to 191)	146 (99 to 198)	124 (79 to 167)
Baltic Sea Diet Score (0–18)	12.0 (9.0 to 15.0)	13.0 (10 to 16.0)	11.0 (9.0 to 14.0)
Device-assessed lifestyle behaviors			
Sleep duration, mean (SD), h/night	9 (1)	9 (1)	9 (1)
Sedentary time, mean (SD), min/d	606 (134)	614 (132)	600 (137)
Light physical activity, mean (SD), min/d	320 (113)	315 (117)	323 (111)
Moderate to vigorous physical activity, median (IQR), min/d	39 (20 to 68)	36 (9 to 45)	50 (25 to 72)
Vigorous physical activity, median (IQR), min/d	6 (1 to 19)	3 (0 to 10)	9 (1 to 25)
Physical activity energy expenditure, mean (SD), kJ/d	53 (23)	46 (20)	58 (25)

^a^ Data on all variables were available for 187 participants except data on device-assessed movement behaviors, which were available for 170 participants.

^b^ Calculated as weight in kilograms divided by height in meters squared.

### Associations of Cumulative Self-Reported Lifestyle Behaviors From Childhood to Adolescence With Mental Health Symptoms in Adolescence

Total PA and supervised exercise were inversely associated with perceived stress and depressive symptoms scores (Table 2). Total ST, computer use, and mobile device use were positively associated with the perceived stress score. Total ST and mobile device use were positively associated with depressive symptoms scores. Diet quality was not associated with mental health scores. The association between total PA and perceived stress scores was no longer statistically significant after further adjustment for BF% at 8-year follow-up (β = −0.15; 95% CI, −0.29 to 0.00). Adjustment for BF% did not affect the magnitude of other associations. Most associations of self-reported PA with mental health symptom scores were attenuated after further adjustment for measures of ST (see eResults in Supplement 1).

**Table 2.** Associations of Cumulative Lifestyle Behaviors From Childhood to Adolescence With Mental Health Symptoms in Adolescence

Self-reported lifestyle behaviors	Perceived stress score		Depressive symptoms score	
	β (95% CI)^a^	*P* value	β (95% CI)^a^	*P* value
Total physical activity, min/d	−0.15 (−0.30 to −0.01)	.04	−0.17 (−0.31 to −0.02)	.03
Unsupervised physical activity, min/d	−0.09 (−0.24 to 0.05)	.21	−0.14 (−0.29 to 0.01)	.06
Sports, min/d	−0.12 (−0.26 to 0.02)	.10	−0.11 (−0.26 to 0.03)	.13
Supervised exercise, min/d	−0.15 (−0.29 to −0.01)	.04	−0.14 (−0.29 to −0.00)	.05
Total screen time, min/d	0.27 (0.13 to 0.41)	<.001	0.30 (0.15 to 0.44)	<.001
TV watching, min/d	0.10 (−0.04 to 0.24)	.16	0.10 (−0.04 to 0.24)	.16
Computer use, min/d	0.16 (0.01 to 0.30)	.03	0.15 (0.00 to 0.30)	.05
Mobile device use, min/d	0.28 (0.16 to 0.41)	<.001	0.33 (0.19 to 0.46)	<.001
Baltic Sea Diet Score	−0.11 (−0.25 to 0.03)	.11	−0.08 (−0.23 to 0.06)	.25
Device-assessed lifestyle behaviors				
Sedentary time, min/d	−0.04 (−0.18 to 0.11)	.61	0.01 (−0.14 to 0.16)	.89
Light physical activity, min/d	0.02 (−0.14 to 0.18)	.76	0.03 (−0.13 to 0.20)	.68
Moderate to vigorous physical activity, min/d	0.09 (−0.11 to 0.29)	.40	−0.04 (−0.24 to 0.16)	.69
Vigorous physical activity, min/d	0.11 (−0.11 to 0.32)	.34	−0.04 (−0.26 to 0.18)	.74
Physical activity energy expenditure, min/d	0.11 (−0.07 to 0.28)	.23	−0.01 (−0.19 to 0.17)	.90
Sleep duration, min/d	−0.09 (−0.25 to 0.06)	.24	−0.08 (−0.24 to 0.07)	.29

^a^ Data are standardized regression coefficients (β) and their 95% CIs adjusted for age, sex, and parental education. Data on all variables were available for 187 participants except that data on device-assessed movement behaviors were available for 170 participants.

Total PA (β = −0.36; 95% CI, −0.55 to −0.16) and unsupervised PA (β = −0.28; 95% CI, −0.47 to −0.08) were inversely associated with perceived stress in boys but not in girls (β = 0.17; 95% CI, −0.05 to 0.38; *P* = .004 for sex × total PA interaction; β = −0.16; 95% CI, −0.06 to 0.38; *P* = .01 for sex × unsupervised PA interaction). Further adjustments had no effect on these associations or interactions.

### Associations of Cumulative Device-Assessed Lifestyle Behaviors From Childhood to Adolescence With Mental Health Symptoms in Adolescence

PA, SB, and sleep duration showed no association with perceived stress or depressive symptoms scores (Table 2). Light PA was positively associated with the perceived stress score (β = 0.22; 95% CI, 0.01 to 0.43) and the depressive symptoms score (β = 0.25; 95% CI, 0.04 to 0.46) in boys. In girls, light PA was not associated with the perceived stress score (β = −0.17; 95% CI, −0.40 to 0.06; *P* = .01 for sex × light PA interaction) or the depressive symptom score (β = −0.13; 95% CI, −0.36 to 0.09; *P* = .02 for sex × light PA interaction). Further adjustments had no effect on these associations or interactions.

## Discussion

Adolescents who reported higher total and supervised PA levels and lower ST time and mobile device use from childhood to adolescence had lower levels of perceived stress and depressive symptoms. The magnitude of most associations of PA with mental health symptoms attenuated after accounting for total ST and mobile device use. Moreover, adolescents who had lower total or supervised PA levels and more total ST or mobile device use from childhood to adolescence had the highest levels of perceived stress and depressive symptoms. The magnitude of most associations was small, but the magnitude of the associations of total screen time and mobile device use with depressive symptoms were considered moderate.

In line with the previous studies in children and adolescents,^45^ self-reported PA, rather than device-assessed PA, from childhood to adolescence was inversely associated with mental health symptoms in adolescence. In boys, self-reported total PA and unsupervised PA were inversely associated with mental health symptoms. Surprisingly, however, device-assessed light PA was positively associated with mental health symptoms in boys. The reason for the negative association between light PA and mental health symptoms remains unclear. Light PA primarily consists of activities such as walking to school, which is prevalent among Finnish children. However, such activities may not provide opportunities to enhance self-esteem or receive positive feedback, both of which are plausible mechanisms underlying the effects of PA on mental health.^5,46^ In general, our results align with the existing literature, suggesting an inverse association between self-reported PA and depressive symptoms in adolescents.^47,48^

Although some studies have suggested an inverse relationship between device-assessed PA and mental health symptoms,^49^ the evidence remains mixed.^45^ Our findings aligned with previous studies, suggesting that supervised exercise may be more important than the volume or intensity of PA in alleviating mental health symptoms.^5,45,50^ However, accounting for self-reported total screen time and mobile device use in the analyses attenuated most associations between PA and mental health. These findings indicate that reducing ST may be more important in promoting mental health than increasing PA.

We found consistent and independent direct associations of cumulative total ST and mobile device use from childhood to adolescence with mental health symptoms in adolescence. We demonstrated that adolescents more exposed to total ST, particularly mobile device use from childhood to adolescence, had higher perceived stress and depressive symptoms independent of their PA levels. Moreover, consistent with the results of previous cross-sectional studies,^13^ we found no association between device-assessed SB and mental health symptoms, suggesting that specific modes of SB, such as ST,^18^ may be more detrimental to mental health than total sedentary time. Our results from the post hoc analyses also suggest that adolescents who accumulate more total ST or mobile device use along with less total PA or supervised exercise experience higher perceived stress and depressive symptoms, supporting previous findings that a combination of unhealthy lifestyle behaviors is particularly detrimental to mental health.^24^ However, further studies investigating which combinations of ST and PA are more strongly related to mental health are warranted.

We observed no association of diet quality or sleep duration from childhood to adolescence with mental health in adolescence. Previous studies have suggested that better diet quality is associated with lower depression in adults.^25,51^ Furthermore, enhancing sleep duration and quality has improved mental health among adults,^6^ and a longer sleep duration has been associated with better mental health in children and adolescents.^21^ A possible explanation for our null associations may be relatively good diet quality and sufficient sleep duration of our study participants.

Interventions aimed at reducing substance abuse, dieting, and negative coping strategies, as well as promoting healthy weight, diet, and sleep, have been advocated as fundamental approaches to fostering mental health among children and adolescents.^52^ However, higher levels of ST and social media use have been related to risk factors and underlying psychosocial mechanisms for mental health symptoms, such as poorer sleep, body image, and self-esteem in adolescents,^53^ underscoring screen time as a modifiable risk factor for mental health problems. One further explanation for the positive association between PA and mental health could be improved physical self-esteem, which is considered the greatest potential mechanism for the positive association between PA and mental health.^45,46,54^ Physicians, nurses, psychologists, and other professionals should discuss ST guidelines with children, adolescents, and their families and emphasize a balanced and safe digital environment to prevent mental health problems among youth. Additionally, fostering consistent engagement in PA within supportive social contexts that promote self-esteem may improve mental health during youth.^5^

### Strengths and Limitations

The strengths of our study include the population-based and well-characterized sample of children followed up until adolescence and comprehensive, valid, and reproducible methods to assess PA, SB, sleep duration, diet quality, and mental health symptoms. Another strength of our study is that we assessed PA and SB using self-reports to capture the behavioral context of PA and SB and used a wearable device to capture PA intensity and volume as recommended.^45^

The study also has limitations that need to be considered. The relatively small sample size at the 8-year follow-up reduced statistical power, especially in the sex-stratified analyses. Moreover, due to the missing data across the follow-up, a relatively large number of participants were omitted from the analyses. Although cumulative indices for lifestyle behaviors provide measures of long-term exposure to these behaviors and partly overcome problems related to over-time variability,^36^ they do not fully capture fluctuation in these behaviors between 2-year and 8-year follow-up, potentially resulting in misestimation. Moreover, we did not assess social and individual contextual factors and specific PA characteristics and screen time content. High exposure to social media may be more greatly associated with mental health symptoms than other screen contents.^55^ Yet, the 8-year follow-up commenced in 2015 to 2017, and the availability and content of social media platforms changed remarkably since then. Therefore, our results should be interpreted cautiously and using the time spent in broad categories of PA and ST. We only assessed sleep duration but not sleep latency or sleep quality, which may have distinct associations with mental health. Additionally, the study design did not allow us to draw conclusions on possible causal relationships between lifestyle behaviors and mental health from childhood to adolescence.

## Conclusion

In conclusion, our findings suggest that adolescents who accumulate more total ST and mobile device use from childhood to adolescence have more perceived stress and depressive symptoms in adolescence. Our results also suggest that adolescents accumulating less PA from childhood may have more mental health symptoms, but the results were inconsistent between self-reported and device-assessed PA. It seems that reducing ST among those unengaged in PA and supervised exercise is important to promote mental health. Intervention studies investigating the effects of screen time reduction on mental health are warranted.

## Notes

Supplement 1.
eMethods.

eResults.
**eFigure 1.** Combined associations of total physical activity and screen time behaviors with mental health symptoms **eFigure 2.** Combined associations of supervised exercise and screen time behaviors with mental health symptoms **eTable 1.** Differences in perceived stress and depressive symptoms scores among adolescents with lower or higher levels of self-reported total PA (< or ≥ sex-specific median) and lower or higher **eTable 2.** Differences in perceived stress and depressive symptoms scores among adolescents with lower or higher levels of supervised exercise (< or ≥ sex-specific median) and lower or higher levels of self-reported total ST or mobile device use (< or ≥ sex-specific median) from childhood to adolescence levels of self-reported total ST or mobile device use (< or ≥ sex-specific median) from childhood to adolescence
eReferences.

Supplement 2.
Data Sharing Statement
