# Supplementary material for: Childhood Lifestyle Behaviors and Mental Health Symptoms in Adolescence
Source: JAMA Netw Open. 2025 Feb 14;8(2):e2460012. doi: 10.1001/jamanetworkopen.2024.60012 (PMC11829227; doi:10.1001/jamanetworkopen.2024.60012)
Supplement: Supplement 1. — eMethods. eResults. eFigure 1. Combined associations of total physical activity and screen time behaviors with mental health symptoms eFigure 2. Combined associations of supervised exercise and screen time behaviors with mental health symptoms eTable 1. Differences in perceived stress and depressive symptoms scores among adolescents with lower or higher levels of self-reported total PA (< or ≥ sex-specific median) and lower or higher eTable 2. Differences in perceived stress and depressive symptoms scores among adolescents with lower or higher levels of supervised exercise (< or ≥ sex-specific median) and lower or higher levels of self-reported total ST or mobile device use (< or ≥ sex-specific median) from childhood to adolescence levels of self-reported total ST or mobile device use (< or ≥ sex-specific median) from childhood to adolescence eReferences. [file jamanetwopen-e2460012-s001.pdf]

Supplemental Online Content

Haapala EA, Leppänen MH, Kosola S, et al. Childhood lifestyle behaviors and mental health symptoms in adolescence. *JAMA Netw Open*. 2025;8(2):e2460012.
doi:10.1001/jamanetworkopen.2024.60012

eMethods.

eResults.

eFigure 1. Combined associations of total physical activity and screen time behaviors with mental health symptoms

eFigure 2. Combined associations of supervised exercise and screen time behaviors with mental health symptoms

eTable 1. Differences in perceived stress and depressive symptoms scores among adolescents with lower or higher levels of self-reported total PA (< or ≥ sex-specific median) and lower or higher

eTable 2. Differences in perceived stress and depressive symptoms scores among adolescents with lower or higher levels of supervised exercise (< or ≥ sex-specific median) and lower or higher levels of self-reported total ST or mobile device use (< or ≥ sex-specific median) from childhood to adolescence levels of self-reported total ST or mobile device use (< or ≥ sex-specific median) from childhood to adolescence

eReferences.

This supplemental material has been provided by the authors to give readers additional information about their work.

© 2025 Haapala EA et al. *JAMA Network Open*.

eMETHODS

Study design and participants

We used data from the Physical Activity and Nutrition in Children (PANIC) study, that is an 8-year physical activity and dietary intervention study, and a long-term follow-up study in a general population of children from Kuopio, Finland.[1] We invited 736 children 6–9 years of age who had been registered for the first grade in one of the 16 public schools of the city of Kuopio in the baseline examinations between October 2007 and December 2009. We mailed the invitation to the principal custodians of the children whose addresses we had received from the school agency of the city of Kuopio. Altogether, 512 (70%) children (248 girls, 264 boys, age range 6–9 years) accepted the invitation and participated in the baseline examinations between October 2007 and December 2009. The participants did not differ in sex, age, height-standard deviation score (SDS) or body mass index (BMI)-SDS from all children who started the first grade in the city of Kuopio in 2007–2009. We excluded six children from the study at baseline either owing to their physical disabilities that could hamper participation in the intervention or withdrawal of the families because they had no time or motivation to attend the study. We also excluded data from two children whose parents or caregivers later withdrew their permission to use these data in the study. The final study sample thus included 504 children at baseline. Of them, 438 (87%) attended the 2-year follow-up examinations in 2009–2011, and 277 (55%) attended the 8-year follow-up examinations in 2015–2017.

The Research Ethics Committee of the Hospital District of Northern Savo approved the study protocol in 2006 (Statement 69/2006) and extended its approval until the 8-year follow-up examinations in 2015 (422/2015). The parents or caregivers of the children provided written informed consent, and the children assented to participation. At the 8-year follow-up, the

© 2025 Haapala EA et al. *JAMA Network Open*.

participants reaffirmed their consent. The PANIC study was conducted in accordance with the principles of the Declaration of Helsinki as revised in 2008.

Assessment of lifestyle behaviors at baseline, 2-year follow-up, and 8-year follow-up

Self-reported lifestyle behaviors. We assessed total physical activity, unsupervised physical activity, sports participation (i.e., participation in organized sports training), participation in all supervised exercise (e.g. sports, afterschool exercise clubs, and other supervised/coached exercise hobbies), total screen time, habitual TV viewing time, computer use, and mobile device (e.g., mobile phones or tablet computers) use by the PANIC Physical Activity Questionnaire filled out by the parents together with their children at baseline and 2-year follow-up. At 8-year follow-up, the adolescents filled in the questionnaire themselves. Sedentary behavior and physical activity questionnaires with a similar structure to the PANIC Physical Activity Questionnaire, such as the Youth Physical Activity Questionnaire, have shown good short–term repeatability over four days with an intraclass correlation of 0.86–0.92.[2]

We assessed the consumption of food and drinks and the intake of nutrients using food records.[3] The food records covered 4 predefined and consecutive days, including 2 weekdays and 2 weekend days or 3 weekdays and 1 weekend day. At baseline and 2-year follow-up, a clinical nutritionist instructed the parents to record all food and drinks consumed by their child using household or other measures, such as tablespoons, deciliters, and centimeters. At 8-year follow-up, the adolescents were instructed to record their food and drink consumption by themselves. A clinical nutritionist checked the returned food records with the participants and filled in any missing information. We calculated food consumption and nutrient intake using the Micro Nutrica® dietary analysis software, Version 2.5 (The Social Insurance Institution of Finland, Turku, Finland). The software is based on detailed information about

© 2025 Haapala EA et al. *JAMA Network Open*.

the nutrient content of foods in Finland and other countries. A clinical nutritionist updated the software by adding new food items and products with their actual nutrient content based on new data in the Finnish food composition database or received from the producers. The Baltic Sea Diet Score (BSDS)[4] was used as an indicator of overall diet quality and was calculated using quartiles of consumption of fruit and berries (scored 0–3), vegetables (0–3), high-fiber grain products (0–3), low-fat (<1%) milk (0–3), fish (0–3), red meat and sausages (reversed 0–3), the ratio of polyunsaturated fatty acids to saturated fatty acids (0–3), and total fat intake as a percentage of total energy intake (reversed 0–3) as described previously. BSDS ranged from 0 to 18, with a higher score indicating better overall diet quality.

Device-assessed lifestyle behaviors. We used a uniaxial accelerometer with a built-in heart rate sensor (Actiheart®, CamNtech Ltd., Papworth, UK) attached to the chest via electrocardiogram (ECG) electrodes to assess physical activity, sedentary behavior, and sleep duration. The device was set to record body movement and heart rate in 60-second epochs. The participants were instructed to continue their usual behavior and to wear the monitor during all daily activities, including sleep, shower, sauna, and swimming, as described previously.[5,6] The participants were requested to wear the device continuously for a minimum of 4 consecutive days; 2 on weekdays and 2 days on the weekend, as the activity patterns of school children vary markedly between weekdays and weekends.[7] We accepted sleep, sedentary time, and physical activity data for statistical analyses if there were at least 48 hours of activity recording in weekday and weekend hours that included at least 12 hours from morning (3 am-9 am), noon (9 am-3 pm), afternoon (3 pm-9 pm), and night (9 pm-3 am) to avoid potential bias from over-representing specific times and activities of the days.[8]

Upon retrieving and downloading the data from the device, heart rate data were first corrected for noise.[9] Subsequently, they were individually calibrated with sleeping heart rate and parameters obtained from maximal exercise tests[10,11] performed by the Ergoselect

200 K® electromagnetic bicycle ergometer (Ergoline, Bitz, Germany) and the Cardiosoft® V6.5 Diagnostic System ECG device (GE Healthcare Medical Systems, Freiburg, Germany). Heart rate data were combined with trunk acceleration data in a branched equation model to estimate activity intensity time-series.[12] Monitor non-wear was acknowledged by prolonged 0 acceleration lasting >90 minutes accompanied by non-physiological heart rate, and activity estimates were adjusted during summarization to minimize diurnal bias arising from non-wear. Physical activity energy expenditure was calculated by integrating the intensity time-series, where time distribution of activity intensity was generated by using standard metabolic equivalents (METs) in 0.5 increments. Sleep duration was analyzed from the Actiheart recordings by a trained exercise specialist and confirmed by a physician, where necessary.[6] The time of falling asleep was defined as accelerometer counts decreasing to 0 and heart rate to a plateau level. Waking time was defined as an increase in accelerometer counts and remaining above zero, and the heart rate increasing and remaining above the plateau level. We defined sedentary behavior as time spent in activity ≤1.5 METs excluding sleep and light, moderate, and vigorous physical activity as time spent in activity >1.5 and ≤4.0 METs, >4.0 and ≤7.0 METs, and >7.0 METs, respectively, by defining 1 MET as 71.2 J/min/kg. These cut-offs have been commonly applied in investigations of physical activity among children and youth.[13] Accelerometers with built-in heart rate monitoring capabilities have been found to be more accurate in estimating physical activity energy expenditure than either method alone in children,[14,15] explaining 86% of the variance in physical activity energy expenditure.[15]

Assessment of mental health symptoms at 8-year follow-up

The adolescents reported their perceived stress using the Finnish version of the Cohen's Perceived Stress Scale,[16] a valid measure of perceived stress in youth.[17] The scale contains 10

© 2025 Haapala EA et al. *JAMA Network Open*.

questions, each scoring from 0 to 4. Thus, the scale ranges between 0 and 40 points, and higher scores indicate higher levels of perceived stress.

We assessed depressive symptoms using the Beck's Depression Inventory (BDI),[18] which is a suitable tool for assessing depressive symptoms in adolescents.[19,20] BDI contains 21 questions, each scoring from 0 to 3. The total scores range between 0 and 63 points, with higher scores indicating higher levels of depressive symptoms.

Other assessments at 8-year follow-up

Body weight was measured twice with the participant having fasted for 12 hours, emptied the bladder, and standing in light underwear using a calibrated InBody® 720 bioelectrical impedance device (Biospace, Seoul, South Korea) to an accuracy of 0.1 kg. The mean of these 2 values was used in the analyses. Body height was measured 3 times with the participant standing in the Frankfort plane without shoes using a wall-mounted stadiometer to an accuracy of 0.1 cm. The mean of the nearest 2 values was used in the analyses. BMI was calculated by dividing weight (kg) by height (m) squared. BMI-SDS was calculated based on Finnish reference data.[21] The prevalence of overweight and obesity was defined using the cut-off values provided by Cole and Lobstein.[22] Body fat mass, body fat percentage (BF%), and lean body mass were measured by the Lunar® dual-energy X-ray absorptiometry device (GE Medical Systems, Madison, WI, USA) using a standardized protocol.[23] A research physician assessed pubertal status using a 5-stage scale described by Marshall and Tanner.[24,25] Testicular volume assessed by an orchidometer was used as an indicator of pubertal status in boys and breast development in girls.

© 2025 Haapala EA et al. *JAMA Network Open*.

Computation of the measures of cumulative lifestyle behaviors

We used the area under the curve (AUC) approach for lifestyle behaviors at baseline, 2-year follow-up, and 8-year follow-up to utilize all the data collected over the 8-year period and describe the exposure to lifestyle behaviors from childhood to adolescence.[26] We estimated participant-specific curves for lifestyle behaviors using an additive linear mixed model with cubic splines, which models non-linear effect as a random effect:[27,28]

y_ij = beta_0 + u_i + f(time_ij) + sex_i + e_ij, where f is the cubic spline, u_i the random intercept, beta_0 intercept, sex_i sex-effect, and e_ij error term. The modeling allowed the inclusion of a non-linear effect which was modeled by cubic spline in addition to random intercept for individuals. These AUC determined using the participant-specific curves were interpreted as long-term exposure to lifestyle behaviors similar to Lai et al.[29] and Hakala et al.[30]

Statistical analyses

Statistical analyses were performed using SPSS software, version 28.0.1.1 (IBM Corp. Armonk, NY, USA). The associations of cumulative lifestyle behaviors from childhood to adolescence over 8 years with perceived stress and depressive symptoms scores in adolescence were investigated using linear regression analyses adjusted for age, sex, and parental education in adolescence. The prevalence and incidence of mental health symptoms[31,32] and the associations between lifestyle behaviors and mental health vary between girls and boys,[33,34] Therefore, we studied the modifying effect of sex on the associations of lifestyle behaviors with perceived stress and depressive symptoms scores, we included a sex x lifestyle behavior interaction term in the models. As pubertal development[35] and body adiposity[36] have been considered important determinants of mental health in youth, the data were additionally adjusted for pubertal status and BF% at 8-year follow-up. The data

© 2025 Haapala EA et al. *JAMA Network Open*.

on statistically significant associations were also mutually adjusted for other cumulative lifestyle behaviors. These measures were entered into the models separately to allow us to quantify their independent influence on the associations. We also adjusted the data for intervention vs. control to test whether the intervention influenced the associations. As it has no effect on the associations, it was not adjusted for in the final analyses. The data were reported as standardized regression coefficients (β), their 95% confidence intervals (CI), and corresponding p-values. β was calculated using the current study population as $\beta = \frac{b \cdot \sigma X}{\sigma Y}$ where b is the unstandardized regression coefficient (slope) from the regression equation, σX is the standard deviation of the predictor variable (X), and σY is the standard deviation of the dependent variable (Y). We considered β of 0.10–0.29, 0.30–0.49, and ≥0.50 to describe the small, moderate, and strong magnitude of the associations, respectively.[37]

As a post hoc analysis based on the strongest predictors of mental health symptoms in the linear regression analyses, we divided adolescents into four groups:

1) lower levels of self-reported total physical activity (< sex-specific median) and lower levels of self-reported total screen time (< sex-specific median) from childhood to adolescence.

2) lower levels of self-reported total physical activity (< sex-specific median) and higher levels of self-reported total screen time (≥ sex-specific median from childhood to adolescence.

3) higher levels of self-reported total physical activity (≥ sex-specific median) and lower levels of self-reported total screen time (< sex-specific median) from childhood to adolescence.

© 2025 Haapala EA et al. *JAMA Network Open*.

4) higher levels of self-reported total physical activity (≥ sex-specific median) and higher levels of self-reported total screen time (≥ sex-specific median) from childhood to adolescence.

Similar groupings were performed using supervised exercise and mobile device use. The differences in perceived stress and depressive symptoms scores between participants in the groups were investigated using General Linear Models adjusted for age, sex, and parental education and considering the Sidak correction for multiple comparisons. All statistical tests were two-tailed.

eRESULTS

Characteristics of participants

The adolescents who did not participate in the 8-year follow-up did not differ in sex, age, pubertal status, height, weight, or BMI-SDS at baseline or 2-year follow-up from those who participated in the 8-year follow-up (all $p > 0.10$ for the difference). Those who did not participate in the 8-year follow-up assessments had higher body fat percentage (BF%) at baseline (mean difference 2.4, 95% confidence interval [CI] 0.8–4.0) and 2-year follow-up (mean difference 2.8, 95% CI 0.9–4.6) and were more likely to be from families without parents with university-level education at baseline (27% vs 43%; $p < 0.001$ at baseline; 31% vs. 43%; $p <0.027$ at 2-year follow-up) than those who participated in the 8-year follow-up.

The associations of self-reported total physical activity and supervised exercise with mental health symptoms after adjustment for measures of screen time

Further adjustment for total screen time and computer use attenuated the associations of total physical activity (β=-0.098, 95CI=-0.242 to 0.045 and β=-0.111, 95% CI=-0.263 to 0.042, respectively) and supervised exercise (β=-0.095, 95% CI=-0.233 to 0.043 and β=-0.116, 95% CI=-0.259 to 0.026, respectively) with the perceived stress score. In addition, adjustment for

© 2025 Haapala EA et al. *JAMA Network Open*.

mobile device use weakened the association between supervised exercise and the perceived stress score (β=-0.121, 95% CI=-0.255 to 0.013).

Further adjustment for mobile device use and total screen time attenuated the associations of supervised exercise with the depressive symptoms score (β=-0.113, 95% CI=-0.249 to 0.023 and β=-0.086, 95% CI=-0.227 to 0.055, respectively). Moreover, adjustment for total screen time attenuated the association between total physical activity and the depressive symptoms score (β=-0.108, 95% CI=-0.254 to 0.039).

Combined associations of cumulative lifestyle behaviors from childhood to adolescence with mental health symptoms in adolescence

Adolescents with lower levels of total physical activity and higher levels of screen time (e**Figure 1A, eTable 1**) or mobile device use (**Figure 1B, eTable 1**) had higher perceived stress and depressive symptoms scores than those with higher levels of total physical activity and lower levels of screen time or mobile device use after adjustment for age, sex, and parental education.

© 2025 Haapala EA et al. *JAMA Network Open*.

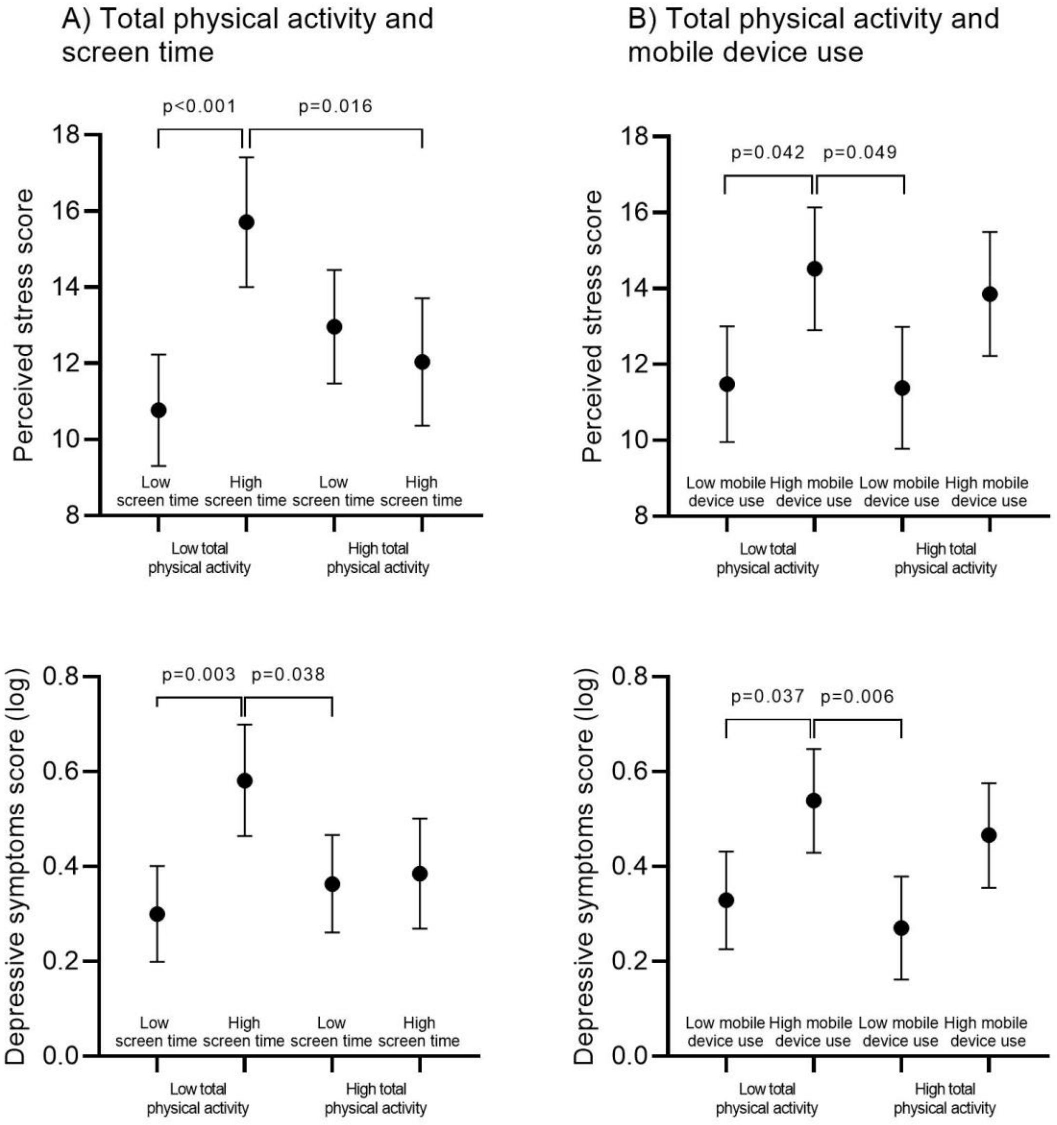

eFigure 1. Combined associations of total physical activity and screen time behaviors with mental health symptoms.

Differences in perceived stress and depressive symptoms scores between adolescents with lower (<sex-specific median) or higher (≥sex-specific median) levels of total physical activity and lower (<sex-specific median) or higher (≥sex-specific median) levels of total screen time (A) or mobile device use (B) from childhood to adolescence. The data were analyzed using General Linear Models adjusted for age, sex, and parental education and considering the Sidak correction for multiple comparisons.

© 2025 Haapala EA et al. *JAMA Network Open*.

Adolescents with lower levels of supervised exercise and higher levels of total screen time (**Figure 2A, eTable 2**) or mobile device use (**Figure 2B, eTable 2**) had higher perceived stress and depressive symptoms scores than those with higher levels of supervised exercise and lower levels of total screen time or mobile device use after adjustment for age, sex, and parental education.

© 2025 Haapala EA et al. *JAMA Network Open*.

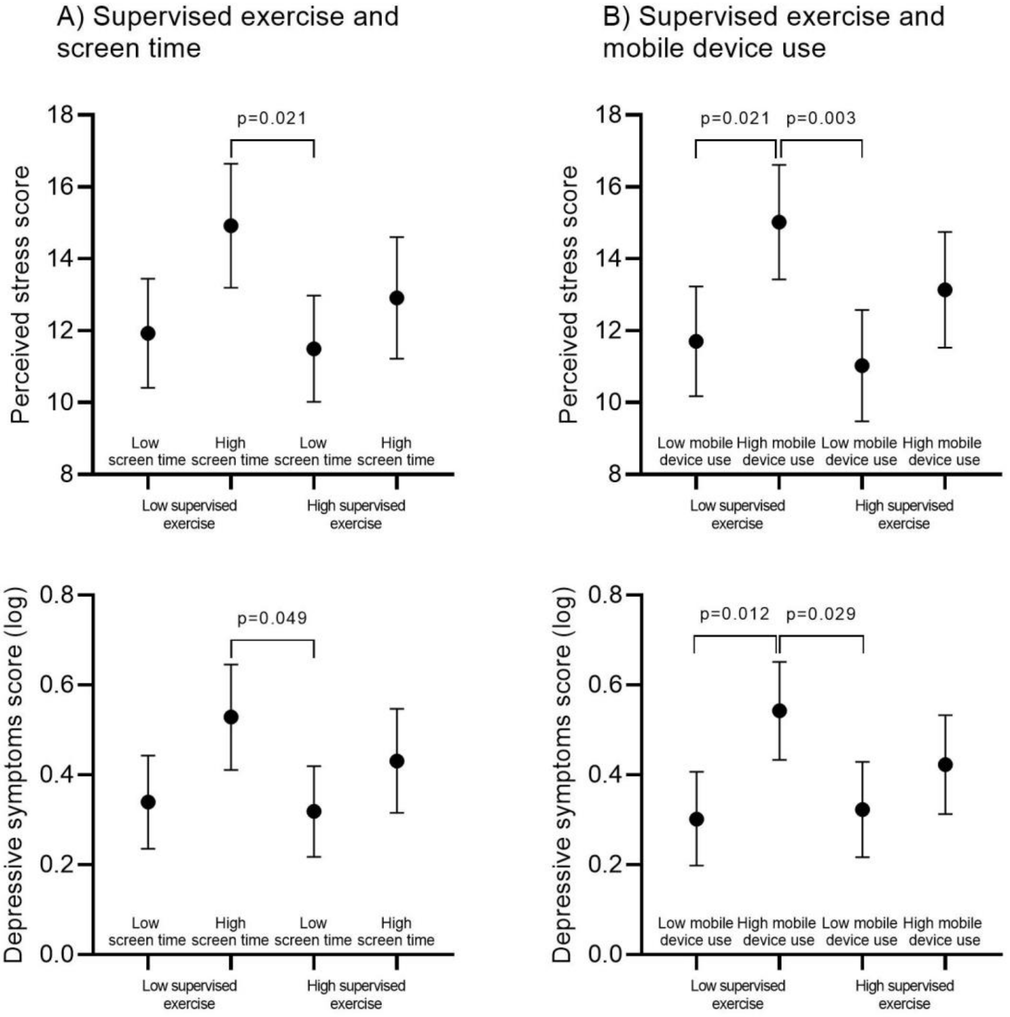

eFigure 2. Combined associations of supervised exercise and screen time behaviors with mental health symptoms.

Differences in perceived stress score and depressive symptoms score between adolescents with lower (<sex-specific median) or higher (≥sex-specific median) levels of supervised exercise and lower (<sex-specific median) or higher (≥sex-specific median) levels of total screen time (A) or mobile device use (B). The data were analyzed using General Linear Models adjusted for age, sex, and parental education and considering the Sidak correction for multiple comparisons.

© 2025 Haapala EA et al. *JAMA Network Open*.

eTable 1. Differences in perceived stress and depressive symptoms scores among adolescents with lower or higher levels of self-reported total PA (< or ≥ sex-specific median) and lower or higher levels of self-reported total ST or mobile device use (< or ≥ sex-specific median) from childhood to adolescence.

	**Perceived stress score**	**Depressive symptoms score (log)**
**Total physical activity and screen time**		
Low total physical activity / low screen time	10.8 (9.3 to 12.2)	0.3 (0.2 to 0.4)
Low total physical activity / high screen time	15.7 (14.0 to 17.4)	0.5 (0.4 to 0.6)
High total physical activity / low screen time	13.0 (11.5 to 14.5)	0.3 (0.2 to 0.4)
High total physical activity / high screen time	12.0 (10.4 to 13.7)	0.5 (0.4 to 0.6)
**Total physical activity and mobile device use**		
Low total physical activity / low mobile device use	11.5 (10.0 to 13.0)	0.3 (0.2 to 0.4)
Low total physical activity / high mobile device use	14.5 (12.9 to 16.1)	0.5 (0.4 to 0.6)
High total physical activity / low mobile device use	11.4 (9.8 to 13.0)	0.3 (0.2 to 0.4)
High total physical activity / high mobile device use	13.9 (12.2 to 15.5)	0.5 (0.4 to 0.6)

Data are means and their 95% confidence intervals. The data were analyzed using General Linear Models adjusted for age, sex, and parental education and considering the Sidak correction for multiple comparisons.

© 2025 Haapala EA et al. *JAMA Network Open*.

eTable 2. Differences in perceived stress and depressive symptoms scores among adolescents with lower or higher levels of supervised exercise (< or ≥ sex-specific median) and lower or higher levels of self-reported total ST or mobile device use (< or ≥ sex-specific median) from childhood to adolescence.		
	Perceived stress score	Depressive symptoms score (log)
**Supervised exercise and screen time**		
Low supervised exercise / low screen time	11.9 (10.4 to 13.5)	0.3 (0.2 to 0.4)
Low supervised exercise / high screen time	14.9 (13.2 to 16.6)	0.5 (0.4 to 0.6)
High supervised exercise / low screen time	11.5 (10.0 to 13.0)	0.3 (0.2 to 0.4)
High supervised exercise / high screen time	12.9 (11.2 to 14.6)	0.4 (0.3 to 0.5)
**Total physical activity and mobile device use**		
Low supervised exercise / low mobile device use	11.7 (10.2 to 13.2)	0.3 (0.2 to 0.4)
Low supervised exercise / high mobile device use	15.0 (13.4 to 16.6)	0.5 (0.4 to 0.7)
High supervised exercise / low mobile device use	11.0 (9.5 to 12.6)	0.3 (0.2 to 0.4)
High supervised exercise / high mobile device use	13.1 (11.5 to 14.7)	0.4 (0.3 to 0.5)

Data are means and their 95% confidence intervals. The data were analyzed using General Linear Models adjusted for age, sex, and parental education and considering the Sidak correction for multiple comparisons.

© 2025 Haapala EA et al. *JAMA Network Open*.

eREFERENCES

1. Lakka TA, Lintu N, Väistö J, et al. A 2 year physical activity and dietary intervention attenuates the increase in insulin resistance in a general population of children: the PANIC study. *Diabetologia*. 2020;63(11):2270-2281

2. Corder K, van Sluijs EM, Wright A, Whincup P, Wareham NJ, Ekelund U. Is it possible to assess free-living physical activity and energy expenditure in young people by self-report? *Am J Clin Nutr*. 2009;89(3):862-870.

3. Sallinen T, Viitasalo A, Lintu N, et al. The effects of an 8-year individualised lifestyle intervention on food consumption and nutrient intake from childhood to adolescence: the PANIC Study. *J Nutr Sci*. 2022;11:e40.

4. Kanerva N, Kaartinen NE, Schwab U, Lahti-Koski M, Männistö S. The Baltic Sea Diet Score: a tool for assessing healthy eating in Nordic countries. *Public Health Nutr*. 2014;17(8):1697-1705.

5. Brage S, Brage N, Franks PW, Ekelund U, Wareham NJ. Brage S, Brage N, Franks PW, Ekelund U, Wareham NJ. Reliability and validity of the combined heart rate and movement sensor Actiheart. Eur J Clin Nutr 59, 561-570. *Eur J Clin Nutr*. 2005;59:561-570.

6. Collings PJ, Westgate K, Väistö J, et al. Cross-Sectional Associations of Objectively-Measured Physical Activity and Sedentary Time with Body Composition and Cardiorespiratory Fitness in Mid-Childhood: The PANIC Study. *Sports Med*. 2017;47(4):769-780.

7. Brooke HL, Corder K, Atkin AJ, van Sluijs EMF. A Systematic Literature Review with Meta-Analyses of Within- and Between-Day Differences in Objectively Measured Physical Activity in School-Aged Children. *Sports Med*. 2014;44(10):1427-1438.

8. Brage S, Westgate K, Wijndaele K, Godinho J, Griffin S, Wareham N. Evaluation of a method for minimising diurnal information bias in objective sensor data. Int Conf Amb Mon Phys Act Mov. 2013 (Conference Proceeding).

9. Stegle O, Fallert SV, MacKay DJC, Brage S. Gaussian Process Robust Regression for Noisy Heart Rate Data. *IEEE Trans Bio Med Eng*. 2008;55(9):2143-2151. doi:10.1109/TBME.2008.923118

10. Brage S, Ekelund U, Brage N, et al. Hierarchy of individual calibration levels for heart rate and accelerometry to measure physical activity. *J Appl Physiol*. 2007;103(2):682-692.

11. Lintu N, Viitasalo A, Tompuri T, et al. Cardiorespiratory fitness, respiratory function and hemodynamic responses to maximal cycle ergometer exercise test in girls and boys aged 9–11 years: the PANIC Study. *Eur J Appl Physiol*. 2015;115(2):235-243.

12. Brage S, Brage N, Franks PW, et al. Branched equation modeling of simultaneous accelerometry and heart rate monitoring improves estimate of directly measured physical activity energy expenditure. *J Appl Physiol*. 2004;96(1):343-351.

13. Janssen I, LeBlanc AG. Systematic review of the health benefits of physical activity and fitness in school-aged children and youth. *Int J Behav Nutr Physl Act*. 2010;7(1):40.

14. Corder K, Brage S, Mattocks C, et al. Comparison of Two Methods to Assess PAEE during Six Activities in Children. *Med Sci Sports Exerc*. 2007;39(12):2180-2188.

© 2025 Haapala EA et al. *JAMA Network Open*.

15. Corder K, Brage S, Wareham NJ, Ekelund U. Comparison of PAEE from Combined and Separate Heart Rate and Movement Models in Children. *Med Sci Sports Exerc*. 2005;37(10):1761.

16. Cohen S, Kamarck T, Mermelstein R. A Global Measure of Perceived Stress. *J Health Soc Behav*. 1983;24(4):385-396. doi:10.2307/2136404

17. Liu X, Zhao Y, Li J, Dai J, Wang X, Wang S. Factor Structure of the 10-Item Perceived Stress Scale and Measurement Invariance Across Genders Among Chinese Adolescents. *Front Psychol*. 2020;11.

18. Beck AT, Ward CH, Mendelson M, Mock J, Erbaugh J. An Inventory for Measuring Depression. *Archives of General Psychiatry*. 1961;4(6):561-571.

19. Bennett DS, Ambrosini PJ, Bianchi M, Barnett D, Metz C, Rabinovich H. Relationship of Beck Depression Inventory factors to depression among adolescents. *J Affect Disord*. 1997;45(3):127-134.

20. Stockings E, Degenhardt L, Lee YY, et al. Symptom screening scales for detecting major depressive disorder in children and adolescents: A systematic review and meta-analysis of reliability, validity and diagnostic utility. *J Affect Disord*. 2015;174:447-463.

21. Saari A, Sankilampi U, Hannila ML, Kiviniemi V, Kesseli K, Dunkel L. New Finnish growth references for children and adolescents aged 0 to 20 years: Length/height-for-age, weight-for-length/height, and body mass index-for-age. *Ann Med*. 2011;43(3):235-248.

22. Cole TJ, Lobstein T. Extended international (IOTF) body mass index cut-offs for thinness, overweight and obesity. *Pediatr Obes*. 2012;7(4):284-294.

© 2025 Haapala EA et al. *JAMA Network Open*.

23. Tompuri TT, Lakka TA, Hakulinen M, et al. Assessment of body composition by dual-energy X-ray absorptiometry, bioimpedance analysis and anthropometrics in children: the Physical Activity and Nutrition in Children study. *Clin Physiol Funct Imaging*. 2015;35(1):21-33.

24. Marshall WA, Tanner JM. Variations in pattern of pubertal changes in girls. *Arch Dis Child*. 1969;44(235):291-303.

25. Marshall WA, Tanner JM. Variations in the Pattern of Pubertal Changes in Boys. *Arch Dis Child*. 1970;45(239):13-23.

26. Cook NR, Rosner BA, Chen W, Srinivasan SR, Berenson GS. Using the area under the curve to reduce measurement error in predicting young adult blood pressure from childhood measures. *Stat Med*. 2004;23(22):3421-3435. doi:10.1002/sim.1921

27. Lin X, Zhang D. Inference in Generalized Additive Mixed Models by Using Smoothing Splines. *J R Stat Soc Series B Stat Methodol*. 1999;61(2):381-400.

28. Wood S. *Generalized Additive Models: An Introduction with R*. 2nd ed. Chapman & Hall/CRC; 2017.

29. Lai CC, Sun D, Cen R, et al. Impact of Long-Term Burden of Excessive Adiposity and Elevated Blood Pressure From Childhood on Adulthood Left Ventricular Remodeling Patterns: The Bogalusa Heart Study. *J Am Coll Cardiol*. 2014;64(15):1580-1587.

30. Hakala JO, Rovio SP, Pahkala K, et al. Physical Activity from Childhood to Adulthood and Cognitive Performance in Midlife. *Med Sci Sports Exerc*. 2019;51(5):882-890.

© 2025 Haapala EA et al. *JAMA Network Open*.

31. Shorey S, Ng ED, Wong CHJ. Global prevalence of depression and elevated depressive symptoms among adolescents: A systematic review and meta-analysis. *Br J Clin Psychol*. 2022;61(2):287-305.

32. Gyllenberg D, Marttila M, Sund R, et al. Temporal changes in the incidence of treated psychiatric and neurodevelopmental disorders during adolescence: an analysis of two national Finnish birth cohorts. *Lancet Psychiatry* 2018;5(3):227-236.

33. Twenge JM, Farley E. Not all screen time is created equal: associations with mental health vary by activity and gender. *Soc Psychiatry Psychiatr Epidemiol*. 2021;56(2):207-217.

34. Haapala EA, Leppänen MH, Skog H, et al. Childhood Physical Fitness as a Predictor of Cognition and Mental Health in Adolescence: The PANIC Study. *Sports Med*. Published online September 10, 2024. doi:10.1007/s40279-024-02107-z

35. Vijayakumar N, Whittle S. A systematic review into the role of pubertal timing and the social environment in adolescent mental health problems. *Clin Psychol Rev* 2023;102:102282.

36. Nwosu E, Makwambeni P, Herstad SH, et al. Longitudinal relationship between adolescents' mental health, energy balance-related behavior, and anthropometric changes. *Obes Rev*. 2023;24(S2):e13629.

37. Cohen J. *Statistical Power Analysis for the Behavioral Sciences*. 2nd ed. L. Erlbaum Associates; 1988.

© 2025 Haapala EA et al. *JAMA Network Open*.
