# Supplementary material for: Childhood Lifestyle Behaviors and Mental Health Symptoms in Adolescence
Source: JAMA Netw Open. 2025 Feb 14;8(2):e2460012. doi: 10.1001/jamanetworkopen.2024.60012 (PMC11829227; doi:10.1001/jamanetworkopen.2024.60012)
Supplement: Supplement 2. — Data Sharing Statement [file jamanetwopen-e2460012-s002.pdf]

Data Sharing Statement

Haapala. Childhood Lifestyle Behaviors and Mental Health Symptoms in Adolescence. *JAMA Netw Open*. Published February 14, 2025. doi:10.1001/jamanetworkopen.2024.60012

Data
Data available: Yes
Data types: Deidentified participant data, Data dictionary
How to access data: The data are not publicly available due to research ethical reasons and because the owner of the data is the University of Eastern Finland and not the research group. The pseudonymised data are available upon reasonable request from the corresponding author (eero.a.haapala@jyu.fi)
When available: With publication

Supporting Documents
Document types: None

Additional Information
Who can access the data: The data are not publicly available due to research ethical reasons and because the owner of the data is the University of Eastern Finland and not the research group. The pseudonymised data are available upon reasonable request from the corresponding author.
Types of analyses: The data will be shared for any purpose.
Mechanisms of data availability: After approval of a proposal with a signed data access agreement.
Any additional restrictions: There are no additional restrictions.
